# Effect of Restricted Feeding on Metabolic Health and Sleep-Wake Rhythms in Aging Mice

**DOI:** 10.3389/fnins.2021.745227

**Published:** 2021-09-07

**Authors:** Yong-Xia Xu, Guo-Ying Liu, Qian Jiang, Han-Qi Bi, Shi-Chan Wang, Ping-Ping Zhang, Chao-Bing Gao, Gui-Hai Chen, Wen-Hui Cheng, Guan-Jun Chen, De-Fa Zhu, Ming-Kui Zhong, Qi Xu

**Affiliations:** ^1^Department of Geriatric Endocrinology, Anhui Geriatric Institute, The First Affiliated Hospital of Anhui Medical University, Hefei, China; ^2^Department of Physiology, School of Basic Medical Sciences, Anhui Medical University, Hefei, China; ^3^Department of Otorhinolaryngology Head and Neck Surgery, The First Affiliated Hospital of Anhui Medical University, Hefei, China; ^4^Department of Sleep Disorders and Neurology, The Affiliated Chaohu Hospital of Anhui Medical University, Hefei, China; ^5^Laboratory Animal Research Center, School of Basic Medical Sciences, Anhui Medical University, Hefei, China; ^6^Center for Scientific Research, Anhui Medical University, Hefei, China

**Keywords:** aging, mice, metabolic symptom, restricted feeding, sleep

## Abstract

Aging, an irreversible and unavoidable physiological process in all organisms, is often accompanied by obesity, diabetes, cardiovascular diseases, sleep disorders, and fatigue. Thus, older adults are more likely to experience metabolic symptoms and sleep disturbances than are younger adults. Restricted feeding (RF) is a dietary regimen aimed at improving metabolic health and extending longevity, as well as reorganizing sleep-wake cycles. However, the potential of RF to improve metabolic health and sleep quality in older adults who are known to show a tendency toward increased weight gain and decreased sleep is unknown. To elucidate this issue, aged mice were assigned to an RF protocol during the active phase for 2 h per day for 2 weeks. Sleep-wake cycles were recorded during the RF regime in RF group and control mice. At the end of this period, body weight and blood biochemistry profiles, including blood glucose, cholesterol, and enzyme activity, in addition to dopamine concentrations in the brain, were measured in the RF group and age-matched controls. RF for 2 weeks improved the metabolic health of aged mice by reducing their body weights and blood glucose and cholesterol levels. At the beginning of the RF regime, sleep decreased in the dark period but not in the light period. After stable food entrainment was achieved (7 days post-RF commencement), the amount of time spent in wakefulness during the light period dramatically increased for 2 h before food availability, thereby increasing the mean duration of awake episodes and decreasing the number of wakefulness episodes. There was no significant difference in the sleep-wake time during the dark period in the RF group, with similar total amounts of wakefulness and sleep in a 24-h period to those of the controls. During the RF regime, dopamine levels in the midbrain increased in the RF group, pointing to its potential as the mechanism mediating metabolic symptoms and sleep-wake regulation during RF. In conclusion, our study suggested that RF during aging might prohibit or delay the onset of age-related diseases by improving metabolic health, without having a severe deleterious effect on sleep.

## Introduction

The circadian system, which is driven by both an internal circadian clock and external environmental signals, controls the timing of feeding, and sleep-wake cycles (Manoogian and Panda, 2017; Northeast et al., 2020). Food scarcity is a major challenge in terms of energy conservation, with prolonged arousal increasing food-seeking behaviors, which indicates reciprocal control between sleep and feeding regulation processes (Arrigoni et al., 2019). Obese individuals and those with metabolic imbalances and diabetes mellitus experience significant disturbances of sleep-wake cycles and sleep architectures (Wu et al., 2014; Koren et al., 2016; Imam et al., 2020). Likewise, sleep deprivation increases the risk of metabolic changes (Knutson et al., 2007; Davies et al., 2014).

Aging is a complex and unavoidable process, which is accompanied by a series of comorbid conditions, including sleep loss, fatigue, metabolic syndrome, and diabetes (Akerstedt et al., 2018; Smith et al., 2020). With increasing age, sleep becomes progressively fragmented and is characterized by impaired consolidation of non-rapid eye movement (NREM) sleep (Klerman and Dijk, 2008; Mander et al., 2017). Age affects homeostatic processes and the number of hours of sleep needed, resulting in a diminished ability to maintain consolidated sleep (Klerman and Dijk, 2008; Mander et al., 2017). Older adults who sleep less show a trend toward an increased body mass index and waist circumference, as well as an increased risk of obesity (Gildner et al., 2014; Grandner et al., 2015; Mamalaki et al., 2019). These results indicate that metabolic symptoms are associated with poor sleep quality during the process of aging.

In contrast to *ad libitum* feeding, where an animal has unrestricted access to food, restricted feeding (RF) is a dietary regime involving fasting for specific periods, which restricts energy intake and appears to reduce body weight (Manoogian and Panda, 2017). Previous research suggested that RF might help to improve sleep quality and quantity and lead to an extended life span (Hou et al., 2019; Oike et al., 2020). In human subjects, 10-h time-restricted eating (TRE) reduced body weight and promoted sleep in obese individuals (Gill and Panda, 2015; Kesztyus et al., 2020). It also improved the cardiometabolic health of patients with metabolic diseases, including controlling blood pressure and decreasing atherogenic lipid cholesterol and glycated hemoglobin (Wilkinson et al., 2020), as well as that of diabetic mice (Hou et al., 2021). Body weight loss via RF may help to lower adiposity, insulin resistance, plasma cholesterol, and plasma triglycerides (Chaix et al., 2014). Based on the aforementioned results from human and animal studies, RF seems to be an effective lifestyle, non-pharmacological intervention to prevent metabolic symptoms and improve health-related quality of life. However, whether RF has beneficial effects on metabolic health and sleep in older adults remains unclear.

The aim of the present study was to investigate the effect of RF on metabolic parameters and sleep-wake architectures. In the current study, aged mice were assigned to an RF protocol for 2 weeks. During this period, their body weights, blood biochemistry profiles, including blood glucose, cholesterol levels, amylopsin, lipase, alanine aminotransferase and alkaline phosphatase activity, total protein, albumin, and globulin levels, and sleep-wake cycles were monitored. The results revealed that RF improved metabolic symptoms and sleep-wake profiles in aging mice, thereby indicating that a non-pharmacological intervention can help to prevent obesity and obesity-associated disorders in the adult population.

## Materials and Methods

### Animals

Male pathogen-free C57BL/6 mice (20–22 months old) were purchased from the Experimental Animal Center of Anhui Province [Certificate No. SCXK (WAN) 2017-006, Hefei, China]. All the mice were maintained under a 12-h light/dark cycle (lights on at 08:00 and off at 20:00) and had access to food and water *ad libitum* prior to the commencement of the study. The maintenance conditions were as follows: ambient temperature of 22 ± 1°C, humidity of 55 ± 5%, and light intensity of ≈ 100 lux (Zhang et al., 2017; Liang et al., 2021). All animal handling procedures and experiments were performed in accordance with the National Institutes of Health Guide for the Care and Use of Laboratory Animals, and all animal experimental procedures were approved by the Animal Care and Use Committee of Anhui Medical University (Wang et al., 2021).

### Electroencephalogram/Electromyogram (EEG/EMG) Electrode Implantation

**FIGURE 1:**
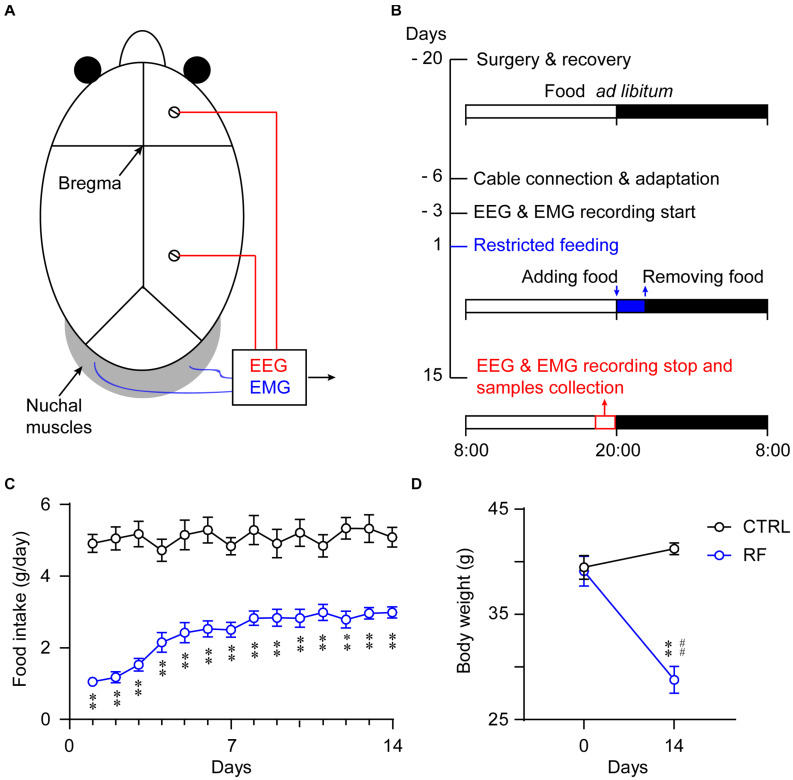
Experimental schedule and measurement of food intake and body weight. Schematic diagram showing the placement of the electrodes for the EEG/EMG recordings **(A)**. Illustration of the RF protocol used in the current experiment. The vertical axis indicates the experimental day, and the horizontal axis represents the manipulation each day. The mice in the RF group had access to food 2 h per day during the 14-day period **(B)**. Food consumption per day of the mice in the RF group and control group (*ad libitum* access to food) during the 14-day period **(C)**. Body weight changes in the RF group versus the control group before and after food restriction **(D)**. ^∗∗^*p* < 0.01 versus CTRL; ^##^*p* < 0.01 versus before food restriction, *n* = 10. CTRL, control; RF, restricted feeding.

The surgical procedure for implanting the EEG/EMG electrodes was as described previously (Xu et al., 2014; Oishi et al., 2017b). Using aseptic techniques, the mice were anesthetized and fixed to a stereotactic frame (RWD Life Science, Shenzhen, China). Two stainless steel screws served as the EEG electrodes, which were inserted into the skull over the right frontal cortex and right parietal cortex. Two insulated Teflon-coated, stainless steel wires (Cooner Wire Co., Los Angeles, CA, United States) placed bilaterally into the neck muscles served as the EMG electrodes (Figure 1A). An implant for sleep-wake recordings was affixed to the skull using self-curing dental cement. The mice were allowed to recover for 14 days after the EEG/EMG electrode implantation surgery.

### RF Protocol

All the mice were kept under a 12-h light/dark cycle (lights on at 08:00 and off at 20:00). After recovery from the EEG/EMG electrode implantation surgery and an acclimation period for 3 days in sleep recording chambers, the mice were allowed free access to food and water for four consecutive days during which EEG/EMG signals were collected as baseline data. The mice were then divided into two groups (an RF group and a control group), and the protocols were applied for 14 consecutive days. The RF group had access to food only for 2 h each day. The same experimenter provided food at 20:00 (lights off/dark period) each night for 14 nights, taking care not to disturb the animals via the use of a low-intensity red light (Zhang et al., 2017) and removed the food at 22:00. The daily food intake was measured by monitoring the food weight before and after feeding for 2 h each day. The control mice received a sham treatment at 20:00, and the food was replaced at 22:00 each day. The food intake in a 24-h interval was calculated as in the RF group. All animals had access to water *ad libitum* throughout the study period (Figure 1B). The body weights of all the mice were recorded before and after the experiment as an indicator of their health status.

### Recording and Scoring of Sleep-Wake Cycles

Fourteen days after recovery from the surgery, each mouse was connected to a cable with a slip-ring for sleep recording in a chamber. This allowed the animal to move freely, without any restrictions during the sleep recordings. All the mice were habituated to the recording cable in a chamber for 3 days before the recordings. The EEG/EMG signals were collected at a sampling rate of 128 Hz (band-pass filter EEG: 0.5–30 Hz, EMG: 20–200 Hz), amplified, and the data were stored on a local computer using Vitalrecorder software (Kissei Comtec, Nagano, Japan). The EEG/EMG data were then scored offline in consecutive 4-s epochs into three vigilance stages: awake, REM sleep, or NREM sleep (SleepSign; Kissei Comtec, Nagano, Japan), in accordance with previously established criteria (Xu et al., 2014; Ren et al., 2018; Cheng et al., 2020; Li et al., 2020). After auto-scoring using SleepSign (Kissei Comtec), the vigilance stage data were checked, and any errors were corrected.

### Blood Sampling and Biochemistry Assay

At the end of the study, blood samples were collected from the two groups between 19:00 and 19:30. The blood samples were collected in a sterilized tube after cardiac puncture (Kube et al., 2021). All the samples were placed on ice for 60 min for blood clotting and then centrifuged at 3,000 × *g* for 10 min, 4°C. The serum samples were then transferred into sterilized tubes for the biochemistry assay. Serum biochemistry profiles, including electrolytes, glucose, amylase, and lipase, were measured using a preloaded Chem 17 CLIP using a Catalyst One analyzer (IDEXX Laboratories, Inc., ME, United States) following the manufacturer’s instructions.

### Brain Tissue Collection and Detection of Dopamine Levels in the Striatum and Midbrain

At the end of the study, all the mice were euthanized and decapitated between 19:00 and 19:30. Striatum and midbrain samples were immediately removed and preserved in ice. They were then homogenized (10% w/v) with phosphate-buffered saline (PBS, 0.01 mol/L, pH 7.4) and centrifuged (3,000 × *g*, 10 min, 4°C). The supernatants were then collected. Dopamine concentrations in the striatum and midbrain were measured using a commercially available dopamine ELISA kit (Cat. # ml002024, Mlbio, Shanghai, China) in accordance with the manufacturer’s instructions.

### Perfusion and Immunohistochemistry

Between 19:00 and 19:30, the mice were anesthetized and perfused transcardially with 30 ml of 0.01 M PBS, followed by 50 ml of chilled 4% paraformaldehyde. The brains were removed and postfixed in 4% paraformaldehyde overnight at 4°C. The samples were then placed in 20% sucrose (dissolved in 0.1 M PBS) at 4°C until sunk to the bottom. The brains were then frozen and coronal slices 30 μm thick were obtained using a freezing microtome (CM1520, Leica, Germany).

During the staining procedure, all the brain slices were kept at room temperature. The brain sections were incubated with the primary antibody for c-Fos (1:1000, Abcam, MA, United States) overnight. The sections were then rinsed with 0.01 M PBS six times and incubated with biotinylated secondary antibody (1:1000, Jackson ImmunoResearch Laboratories, Inc., PA, United States) for 90 min. All the tissue sections were treated with avidin-biotin complex (1:1000, Vector Laboratories, CA, United States) for 60 min, and immunopositive cells were visualized black by reaction with 3,3-diaminobenzidine with nickel (DAB Substrate Kit, Vector Laboratories, CA, United States). After rinsing with PBS, the slices were mounted, dried, dehydrated by ethanol and xylene, and cover slipped. Images were obtained using a BX53 microscope (Olympus, Japan).

### Statistical Analysis

All the results are expressed as the mean ± SEM. Time-course changes in the durations of the sleep-wake stages in the control and RF groups were compared using a two-sided Student’s *t*-test. A two-way repeated-measures analysis of variance, followed by Tukey’s *post hoc* test was conducted to compare body weight changes. In all cases, a value of *p* < 0.05 was taken as the level of statistical significance.

## Results

### RF Reduced the Body Weights of Aging Mice

Food intake in the control group remained unchanged during the study (Figure 1C). In contrast, food intake in the RF group dramatically decreased on the first day of the RF protocol, before progressively increasing and remaining consistent after day 7 (*p* < 0.01, Figure 1C). The mean body weight of the mice in the RF group decreased 30.2% as compared with that before the RF regimen (*p* < 0.01, Figure 1D). There was no significant difference in the body weights of the mice in the control group at the end of the study versus the baseline values (Figure 1D).

### RF Improved Metabolic Symptoms in Aging Mice

**FIGURE 2:**
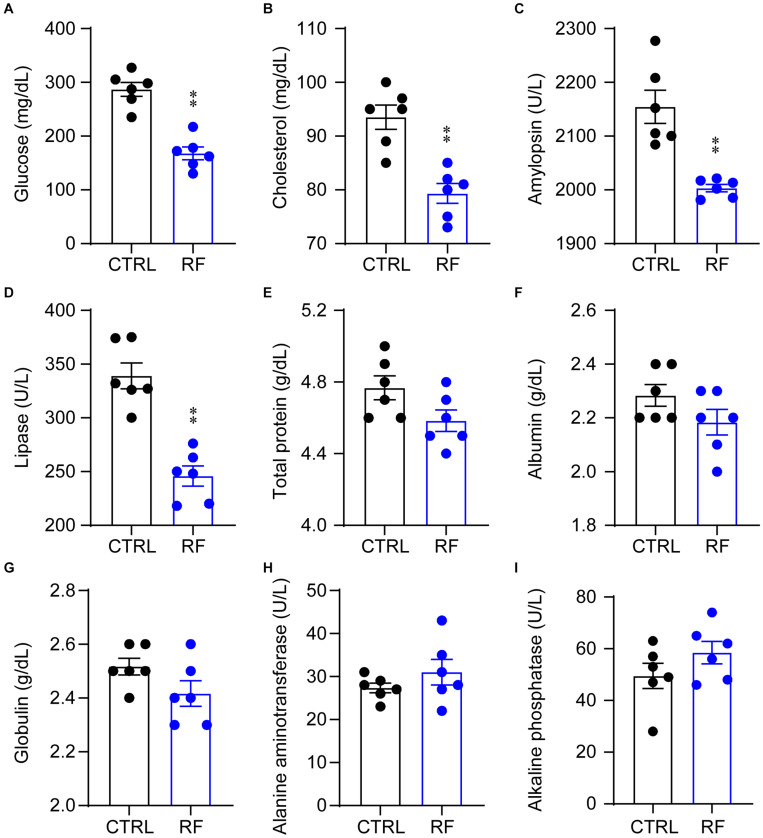
Biochemical parameters in the RF and CTRL groups. Blood glucose **(A)**, cholesterol **(B)**, amylopsin **(C)**, lipase **(D)**, total protein **(E)**, albumin **(F)**, globulin **(G)**, alanine aminotransferase **(H)**, and alkaline phosphatase **(I)**. ^∗∗^*p* < 0.01 versus CTRL, *n* = 6. CTRL, control; RF, restricted feeding.

To determine whether RF improved metabolic symptoms in aging mice, we measured and compared serum biochemical parameters in the control and RF groups. Blood glucose and cholesterol levels significantly decreased in the RF group as compared with those in the control group (*p* < 0.01, Figures 2A,B). In addition, compared to the control group, amylopsin and lipase activity decreased in the RF group (*p* < 0.01, Figures 2C,D). There was no significant between-group difference in total protein, albumin, and globulin levels or alanine aminotransferase and alkaline phosphatase activity (Figures 2E–I). These results indicated that RF improved metabolic symptoms in aging mice by decreasing the levels of blood glucose and cholesterol, without inducing adverse health effects, as serum protein levels and liver function were undamaged by RF.

### Sleep Time Decreased in the RF Group During the Light Period

**FIGURE 3:**
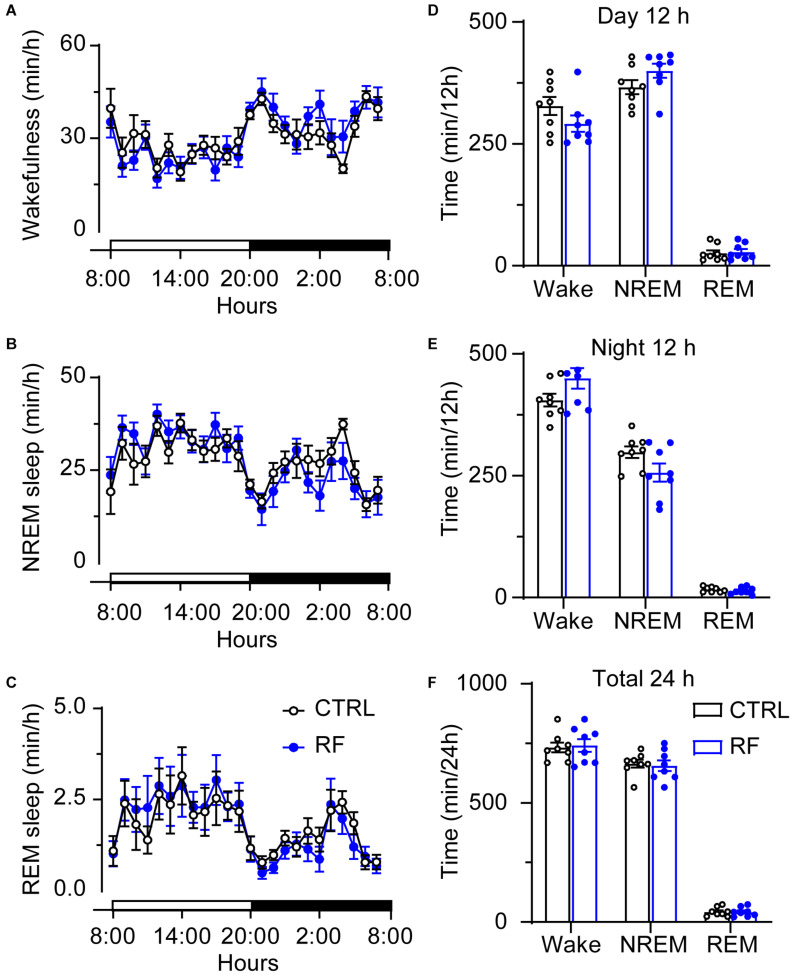
Time spent in sleep-wake stages under baseline conditions of the mice in the RF and CTRL groups. Time spent in wakefulness **(A)**, NREM sleep **(B)**, and REM sleep **(C)** over a 24-h period. Cumulative time of wakefulness, NREM sleep, and REM sleep during the light period **(D)**, dark period **(E)**, and 24-h period **(F)**. Open and closed bars above the *x*-axis indicate light and dark periods, respectively. *n* = 6, CTRL, control; RF, restricted feeding.

**FIGURE 4:**
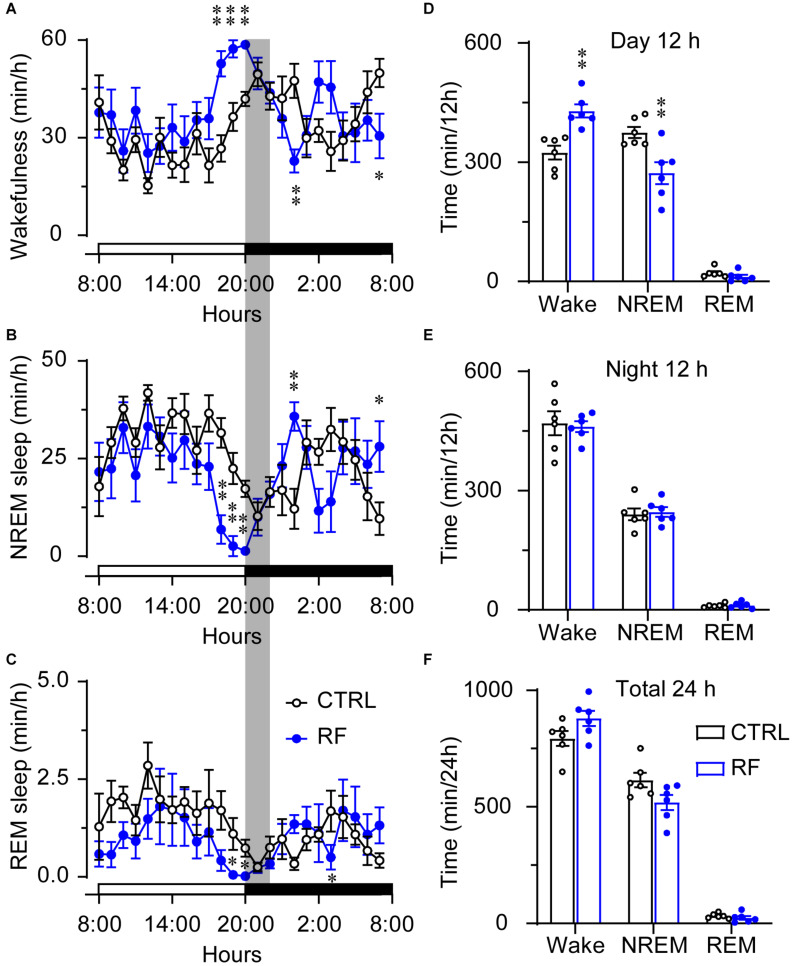
Time the mice in the RF and CTRL groups spent in sleep-wake stages on day 14 of the RF protocol. Time course of wakefulness **(A)**, NREM sleep **(B)**, and REM sleep on day 14 of the RF protocol **(C)**. Cumulative time of wakefulness, NREM sleep, and REM sleep during the light period **(D)**, dark period **(E)**, and 24-h period **(F)**. The gray box indicates food availability during the dark period. Open and closed bars above the *x*-axis indicate light and dark periods, respectively. ^∗^*p* < 0.05, ^∗∗^*p* < 0.01 versus CTRL, *n* = 6. CTRL, control; RF, restricted feeding.

On day 0, before commencement of the RF protocol, there was no significant difference in the sleep-wake profiles of the mice in the RF and control groups, both of which had access to food *ad libitum* (Figures 3A–C). As shown in Figures 3D–F, there was no significant difference in wakefulness, NREM sleep, and REM sleep time during the light and dark periods and total 24-h period between the two groups at baseline. After the mice in the RF group were switched to RF between 20:00 and 22:00 for 14 days, the sleep time progressively decreased compared to that in the controls. On day 3, the mice in the RF group showed an increase in wakefulness and a decrease in NREM sleep and REM sleep, which indicated that the mice spent more time awake in search of food (Supplementary Figures 1A–C). The sum of wakefulness, NREM sleep, and REM sleep revealed that sleep time decreased during the dark and light periods in the RF group (Supplementary Figures 1D–F). From day 7 of the RF protocol, the mice exhibited increased wakefulness [i.e., food anticipation activity (FAA)] 2–3 h prior to the food availability time (*p* < 0.01, Figure 4A), with a reduction in both NREM and REM sleep during this period (*p* < 0.01, Figures 4B,C). Figure 4D shows the wake, REM, and NREM time in the two groups, with a significant between-group difference during the light period (08:00–20:00). There was no significant between-group difference in wakefulness, NREM sleep, and REM sleep during the dark period (Figure 4E) or total 24-h period (Figure 4F). From day 7 of the study, the mice in the RF group showed typical food anticipation behavior (Supplementary Figure 2). When compared to the control mice, these results indicated that RF decreased NREM sleep in the 2-h period prior to food availability and that it had no effect on sleep patterns at any other time during the day.

### RF Altered the Sleep-Wake Architecture in Aging Mice

**FIGURE 5:**
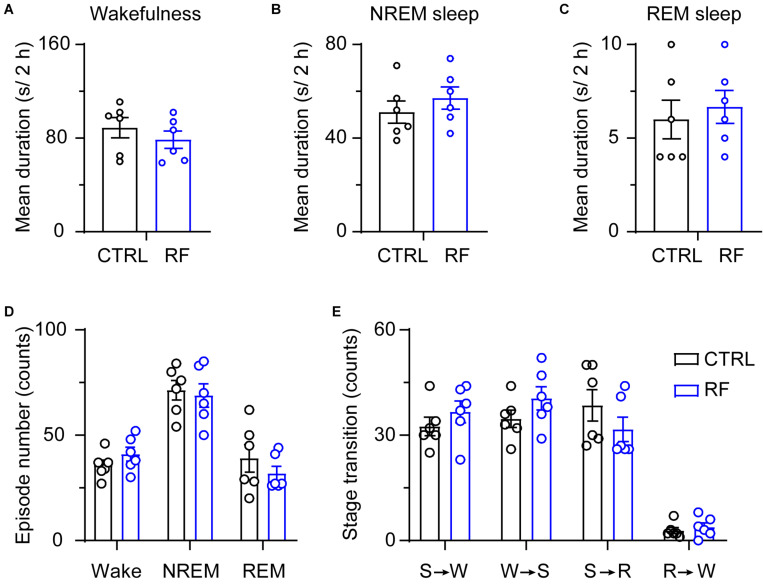
Sleep-wake architecture in the RF and CTRL groups at baseline. The mean durations of wakefulness **(A)**, NREM sleep **(B)**, and REM sleep episodes **(C)** and number of sleep-wake and sleep-wake transition episodes during the 2 h proceeding the dark period **(D)**. Transitions between stages during the 2 h proceeding the dark period **(E)**. CTRL, control; RF, restricted feeding; R, REM sleep; S, slow-wave sleep; W, wakefulness.

**FIGURE 6:**
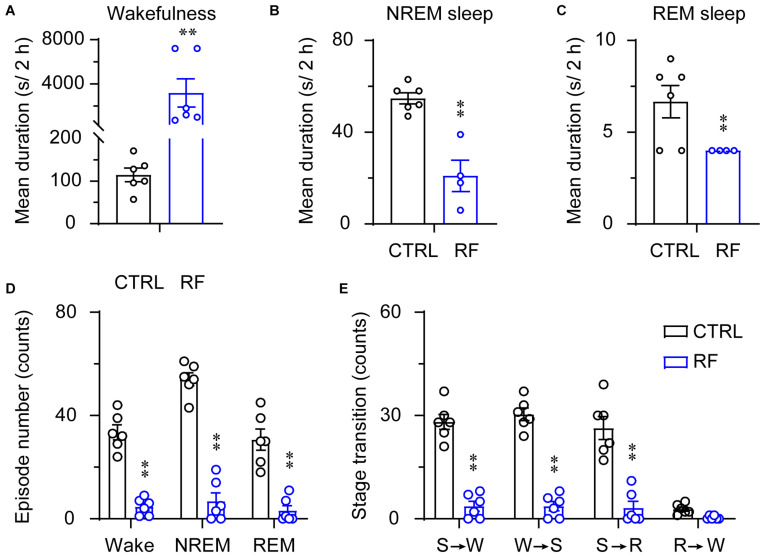
Sleep-wake architecture in the RF and CTRL groups on day 14 of the RF protocol. The mean durations of wakefulness **(A)**, NREM sleep **(B)**, REM sleep **(C)**, and number/type of episode during each stage **(D)** during the 2 h proceeding the dark period (food availability). **(E)** Transitions between stages during the 2 h proceeding the dark period. ^∗∗^*p* < 0.01 versus CTRL, *n* = 6. CTRL, control; RF, restricted feeding; R, REM sleep; S, slow-wave sleep; W, wakefulness.

Under the baseline conditions on day 0, there were no significant changes in the mean duration of sleep-wake episodes or number of sleep-wake transition episodes between 18:00 and 20:00 (Figure 5). After 14 days, during the 2-h period before food availability, the mean duration of wakefulness in the RF group increased (*p* < 0.01, Figure 6A), whereas the mean durations of NREM sleep and REM sleep decreased (*p* < 0.01, Figures 6B,C) compared to those in the control mice. The number of wakefulness, NREM sleep, and REM sleep episodes dramatically decreased in the RF group on day 14 (*p* < 0.01, Figure 6D). RF also decreased the numbers of sleep-wake transition episodes in the RF group compared with those in the control group (*p* < 0.01, Figure 6E). However, during the 10-h light period, there was no significant difference in the mean duration, number of episodes of each stage, and number of sleep-wake transitions before and after RF (Supplementary Figures 3, 4). These findings suggested that RF induces wakefulness by increasing the mean duration of wakefulness episodes and decreasing the number of wakefulness episodes.

### RF Increased the EEG Power Density During NREM and REM Sleep During Food Anticipation in Aging Mice

**FIGURE 7:**
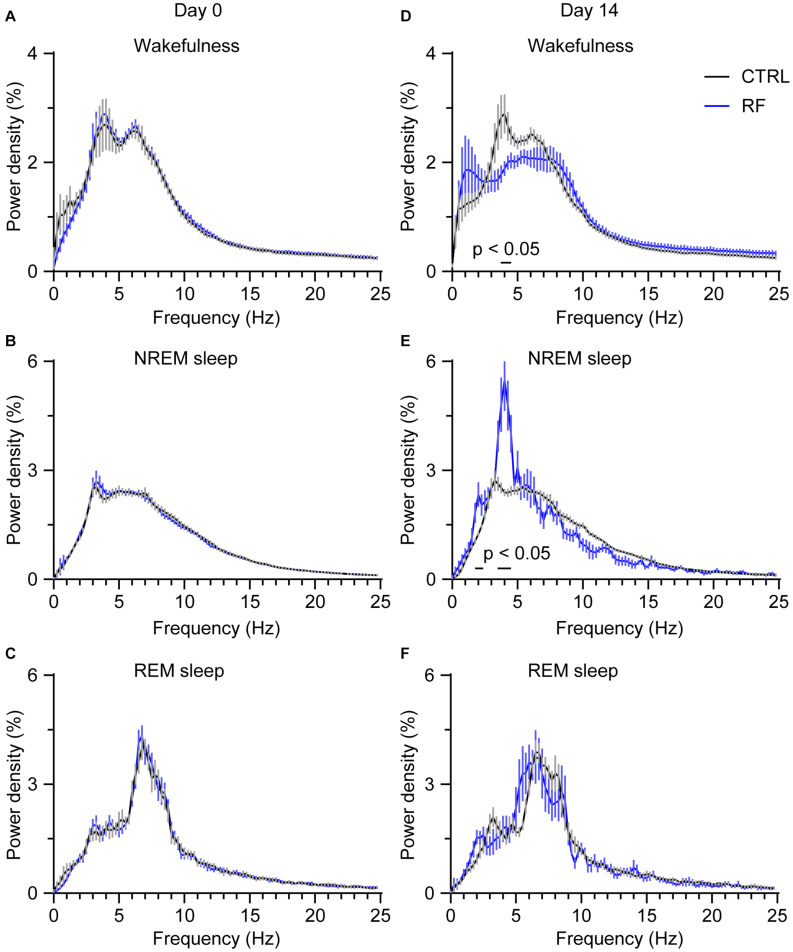
Changes in the EEG power density during wakefulness, NREM sleep, and REM sleep in the 2 h proceeding the dark period (food availability). EEG power density during wakefulness **(A)**, NREM sleep **(B)**, and REM sleep **(C)** in the 2 h proceeding the dark period under the baseline condition (day 0 of the RF regime). EEG power density during wakefulness **(D)**, NREM sleep **(E)**, and REM sleep **(F)** in the 2 h proceeding the dark period (food availability) on day 14 of the RF regime. “The horizontal bars indicate where there is a statistical difference,” versus CTRL (*p* < 0.05).

To assess whether RF altered wake-sleep patterns and sleep quality, we first measured the relative change in EEG power density during the light period. On day 0 of the RF regime, there was no between-group difference in the relative EEG power density during wakefulness, NREM sleep, and REM sleep (Figures 7A–C). After achieving stable food entrainment, a reduction in the wake power spectra within the frequency range of 3.75–4.25 Hz was achieved. However, during the 2 h before food availability, the EEG power density significantly increased during NREM sleep in the RF group compared to that in the control group (*p* < 0.05, Figures 7D–F). In contrast, during the 10 h light period (08:00–18:00), there was no change in the EEG power spectra in the RF group versus that in the control group on either day 0 or day 14 (Supplementary Figures 5, 6). These results pointed to weakening of the arousal state and enhanced sleep pressure during the food anticipation period in the RF regime.

### RF Increased Dopamine Levels in the Midbrain but Not in the Striatum

**FIGURE 8:**
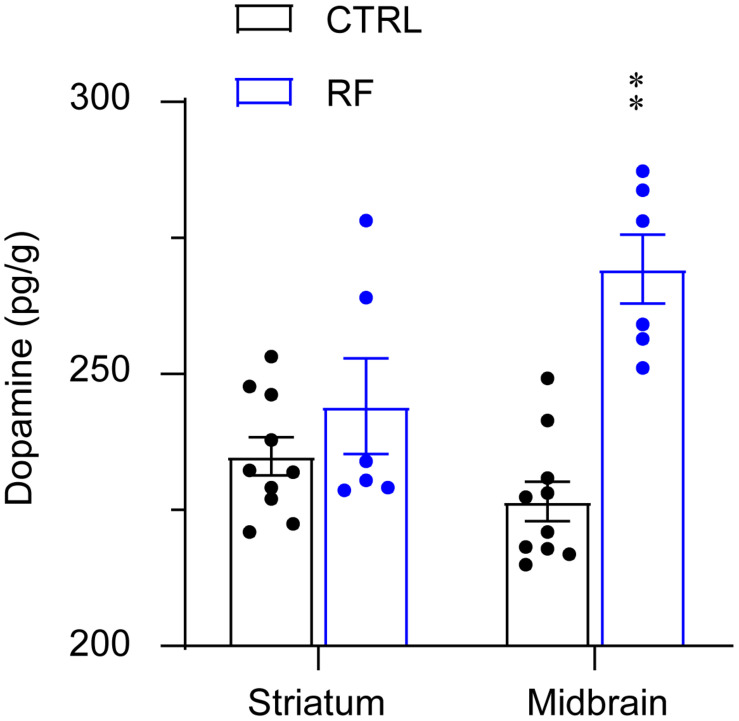
Dopamine concentrations in the striatum and midbrain of the RF and CTRL mice. Dopamine levels in the striatum left panel and midbrain right panel measured by an ELISA. ^∗∗^*p* < 0.01 versus CTRL, *n* = 6–10. CTRL, control; RF, restricted feeding.

The dopamine level in the midbrain significantly increased in the RF group (32%) as compared with that in the control group (*p* < 0.01, Figure 8). Although dopamine levels in the striatum in the RF group showed an increased trend as compared with those in the control group, the finding was not statistically significant. There was no significant difference in the dopamine concentration in the striatum in the RF group versus that in the control group. These results indicated that an increase in dopamine transmission before food availability might contribute to increased wakefulness before feeding.

### RF Induced c-Fos Protein Expression in the Brain

**FIGURE 9:**
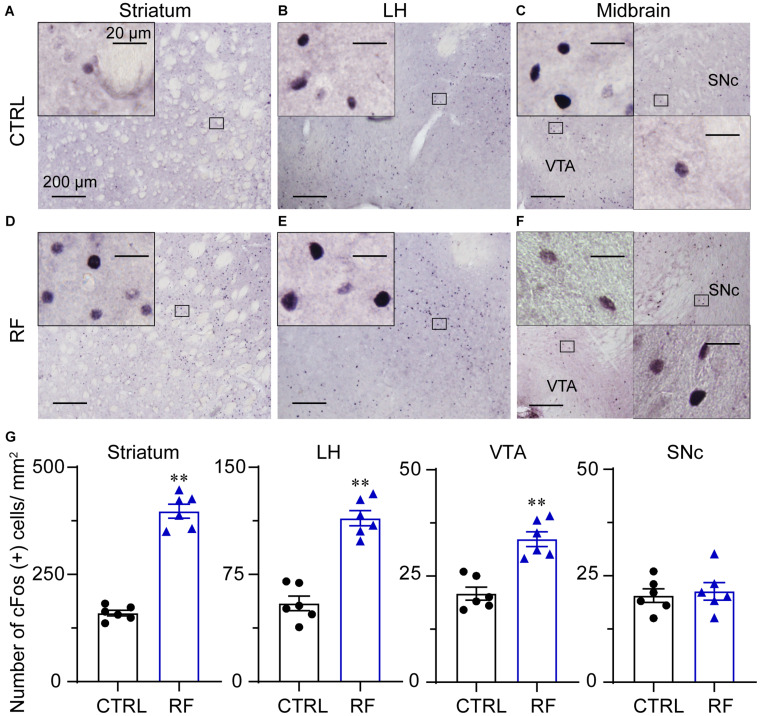
Expression of the c-Fos protein in the RF and CTRL mice. Representative images of c-Fos expression in the striatum **(A,D)**, LH **(B,E)**, and midbrain **(C,F)** of the CTRL and RF mice, respectively. **(G)** Mean numbers of c-Fos-positive neurons in the striatum, LH, VTA, and SNc. ^∗∗^*p* < 0.01 versus CTRL, *n* = 6. CTRL, control; RF, restricted feeding; LH, lateral hypothalamus; VTA, ventral tegmental area; SNc, substantia nigra pars compacta.

To explore the potential mechanism of sleep–wake regulation by RF in aged mice, we analyzed c-Fos protein expression in the brain of the CTRL (Figures 9A–C) and RF (Figures 9D–F) mice. A quantitative analysis revealed an increased number of c-Fos-positive neurons in the striatum (caudate and putamen nuclei), lateral hypothalamus, and ventral tegmental area (*p* < 0.01, Figure 9G). However, there was no significant difference in c-Fos protein expression in the substantia nigra (*p* = 0.71, Figure 9G). These results indicated that many nuclei including midbrain, striatum, and lateral hypothalamus might contribute to RF-mediated wakefulness.

## Discussion

In the current study, we found that RF improved metabolic symptoms by reducing body weights and blood glucose and cholesterol levels. Furthermore, RF led to anticipation of food through increased wakefulness, with reduced sleep. At the beginning of RF, sleep decreased in the RF group in the dark period. After stable food entrainment was achieved around day 7 in the RF group, the amount of time spent in wakefulness in the 2 h prior to food availability (18:00–20:00) increased, thereby increasing the mean duration of awake episodes. There was no significant difference in the amount of wakefulness, NREM sleep, and REM sleep during the dark period in the RF group as compared with that in the control group. During the RF regime, dopamine levels in the midbrain increased in the RF group, suggesting that dopamine transmission in the midbrain might contribute to increased wakefulness during food anticipation.

The incidence of many chronic diseases, including sleep disorders, obesity, cancer, and neurodegenerative disorders, has increased in the aged population (Lopez-Otin et al., 2016). Pharmacological approaches aimed at combating sleep disorders and associated morbidities and improving the quality of life in this population have proved ineffective. Thus, there is increased interest in identifying non-pharmacological strategies. One such approach is intermittent fasting (IF), which involves fasting for extended periods, with intervening periods of normal food intake, on a recurring basis (Mattson et al., 2017). TRE is a subcategory of IF. In TRE, food intake is restricted to a time window of 8 h or less per day. Unlike IF, the focus in TRE is on how many hours per day food consumption is permitted rather than on how long a person must fast (Chaix et al., 2014). The available evidence suggests that the IF protocol produces equivalent weight loss when compared to the TRE regime (Rynders et al., 2019). TRE is very helpful for reducing weight gain linked to late night snacking. In the present study, we chose an RF regime of 2 h during the active phase of mice to test the effects of RF on metabolic health and sleep-wake cycles. At the end of the 14-day period, the mean body weight of the mice in the RF group had decreased 30.2% as compared with the baseline value. Previous studies showed that most body weight loss in RF regimes is due to a decrease in white adipose tissue, especially visceral fat stores (Barzilai et al., 1998; Finkel, 2015; Akbari et al., 2019). In contrast to food restriction, which is thought to slow down the aging process, obesity is believed to exacerbate this process. According to some research, adverse effects of obesity on the function of mitochondria and nutrient-signaling pathways might result in an imbalance in insulin and blood glucose (Bentley et al., 2018). The mechanistic target of rapamycin (mTOR) controls cell growth and metabolism and energy level sensing. Previous studies suggested that mTOR activation promotes cell growth and proliferation by inducing protein synthesis and lipogenesis and increasing glucose metabolism (Jacinto et al., 2004; Hagiwara et al., 2012). In contrast, inhibition of mTOR reduced mRNA translation and protein synthesis (Saxton and Sabatini, 2017) and increased the lifespan (Harrison et al., 2009; Liu and Sabatini, 2020). Food restriction activated adenosine monophosphate-activated protein kinase, which then inhibited and suppressed mTOR signaling to slow aging and increase resistance to pathologies, including metabolic diseases (Burkewitz et al., 2014; Sunjaya and Sunjaya, 2021). These findings are consistent with RF-mediated body weight loss in the aged mice in the present study.

We also found that RF improved cardiometabolic symptoms by lowering blood glucose levels and cholesterol levels in aged mice. Previous studies showed that time RF maintained glucose homeostasis (Mitchell et al., 2015) and lowered fasting blood glucose levels in diabetic mice (Wei et al., 2019). In rodent liver, an RF regime upregulated the metabolic rate, which increased fatty acid β oxidation and reduced hepatic glucose production (Hatori et al., 2012). RF also regulated the metabolic rate by inducing gluconeogenesis and balancing glucose levels. In addition, RF improved the expression of a rate-limiting enzyme, cholesterol 7α-hydroxylase, which promotes bile acid production from cholesterol (Froy and Miskin, 2010; Longo and Panda, 2016; Froy, 2018). We found no significant difference in the serum concentrations of total protein, albumin, and globulin or alkaline phosphatase and alanine aminotransferase activity in the RF group versus the control group. These results indicated that RF can improve cardiometabolic health, without deleterious effects on liver function.

In animal studies, RF during both light and dark periods can lead to food entrainment by increasing locomotor activity (Liu et al., 2012; Caba et al., 2021). In previous studies, when the RF time occurred during the light period in adult animals, the time spent in both NREM and REM sleep decreased, with increased wakefulness (Mieda et al., 2004; Szentirmai et al., 2010; Northeast et al., 2019). In adult mice, when the RF time occurred during the light period, the time spent in sleep-wake stages changed, with increased wakefulness for 2–3 h preceding food availability. However, RF during the light period resulted in a reversal in the light-dark pattern of sleep-wake stages (Mieda et al., 2004). Other studies showed that RF for 4–6 h in the dark period improved sleep-wake cycles in diabetic (Hou et al., 2019) and Huntington’s murine models (Wang et al., 2018). Although these later studies defined sleep in adult animals by immobility, without EEG/EMG recordings, their findings suggested that RF during the active phase has a beneficial effect on circadian rhythms. In the present study, food was provided at 20:00 (lights off) and removed 2 h later, as described previously (Liu et al., 2012). At baseline conditions, when the animals in both the RF and control groups had access to chow and water *ad libitum*, there were no significant differences in the sleep-wake rhythms of the two groups. After the start of the RF protocol, both NREM and REM sleep decreased in the dark period but not in the light period in the RF group as compared with that in control group. Stable food entrainment was achieved in the RF group around 7 days after the commencement of the RF protocol. At this point, wakefulness, characterized by an increased mean duration of wakefulness and a decreased number of wakefulness episodes, dramatically increased during the 2-h period before food availability. We found no significant difference in the sleep-wake cycles in the RF and control groups during the 10-h light (08:00–18:00) and dark periods (20:00–8:00). During RF days 7–14, the sleep-wake profiles in the RF group during each 24-h period were similar, without any significant difference in the mean duration and number of wakefulness, NERM sleep, and REM sleep episodes or any significant difference in the total amount of wakefulness. In contrast, previous studies showed that caloric restriction and fasting increased the amount of wakefulness in rats, with longer term food deprivation (>7 days) causing sleep to become fragmented (Borbely, 1977; Szentirmai et al., 2006; Castro-Faundez et al., 2016). The use of aged mice and a different RF schedule in our study may account for the discord between our findings and those in the literature.

In addition to RF affecting sleep-wake vigilance, RF also altered sleep and wakefulness quality, as quantified by the EEG power density for wakefulness, NREM sleep, and REM sleep. During the 2 h before food availability, the EEG power density for wakefulness under the RF protocol decreased in the frequency of 3.75–4.25 Hz, which was associated with an increased EEG power density in NREM sleep in the frequency bands of 1.75–2.25 Hz and 3.5–4.5 Hz. These observations indicated that the mice in the RF group showed a low inclination for arousal during RF and that both food anticipation and circadian rhythms might regulate sleep-wake cycles. In our study, we detected no significant difference in the EEG power density during the 10-h light period (08:00–18:00) in the RF group compared to that in the control group. Our findings are in accordance with those of studies on wild-type mice and diabetic mice, in which the authors reported that homeostatic regulation of sleep and wakefulness remained intact during an RF protocol (Hou et al., 2019; Northeast et al., 2019).

Previous studies showed RF increased c-Fos protein expression in the dorsomedial nucleus, lateral hypothalamus (LH), perifornical area, and tuberomammillar nucleus, which indicated that FAA may be regulated by networks distributed throughout the brain or that more essential nuclei controlling FAA remain to be unidentified (Angeles-Castellanos et al., 2004). The dopaminergic system, including dopamine and its receptors, is known to be involved in regulating sleep-wake cycles (Qu et al., 2008; Luo et al., 2018) and reward-motivating behavior (Berridge et al., 2010; de Lartigue and McDougle, 2019). As shown in previous studies, genetic depletion of tyrosine hydroxylase or concurrent ablation of dopamine D1 and D2 receptors in mice induces mortality a few weeks after birth. The feeding behavior and physiological function of the gastrointestinal system were altered in these mice, indicating a strong relationship between the dopamine system and gastrointestinal function (Zhou and Palmiter, 1995; Kobayashi et al., 2004; Palmiter, 2007). Previous research also demonstrated that dopamine is crucial for maintaining wakefulness. For example, knockout (KO) of dopamine D2 receptors significantly decreased wakefulness, with an increase in both NREM and REM sleep in mice (Qu et al., 2010). Brain neuronal calcium signaling by dopaminergic neurons in the brain was shown to be higher during wakefulness and lower during sleep, and calcium signals increased significantly during transitions from NREM sleep to wakefulness (Eban-Rothschild et al., 2016; Dong et al., 2019), which indicated that dopaminergic neurons in the midbrain are essential for initiating and maintaining wakefulness (Eban-Rothschild et al., 2016; Oishi et al., 2017a; Yang et al., 2018; Ciric et al., 2019). Studies also showed that dopaminergic midbrain signals govern neuron activity in the striatum and nucleus accumbens to initiate and maintain wakefulness for food seeking (Eban-Rothschild et al., 2016; Luo et al., 2018). The aforementioned findings support the results of our study, in which c-Fos protein expression in the midbrain and striatum and dopamine concentration in the midbrain increased prior to food availability in the RF group.

In addition to dopamine, several other neurotransmitters regulate food intake and sleep and are therefore thought to regulate FAA (Pendergast and Yamazaki, 2018). Pharmacologically increasing serotonin levels by a selective serotonin reuptake inhibitor, fluvoxamine, suppressed FAA, whereas decreasing serotonin levels by a tryptophan hydroxylase inhibitor enhanced FAA in mice (Rozenblit-Susan et al., 2016). However, FAA was unaffected by time RF in both serotonin transporter and tryptophan hydroxylase-2 KO mice, which indicated that serotonin is not necessary for regulating FAA (Gallardo et al., 2020). Some studies suggested that a neuropeptide knows as orexin or hypocretin may be involved in the regulation of food anticipation behavior. Orexin, which strongly promotes food intake and wakefulness, is produced by lateral and posterior hypothalamic neurons (Cun et al., 2014; Soya and Sakurai, 2020; Concetti and Burdakov, 2021). Administration of orexin into the lateral ventricle stimulated food consumption in rodents (Sakurai et al., 1998), and ablation of orexin neurons or KO of the orexin gene in rodents attenuated food intake and obesity (Hara et al., 2005; Fujiki et al., 2006), suggesting that the orexinergic system contributes to food entrainment. The aforementioned findings are consistent with the results of our study, in which c-Fos expression significantly increased in the lateral hypothalamus in the RF group. In another study, FAA was attenuated in orexin KO mice but not eliminated, and core body temperatures and food consumption in the orexin KO mice and control mice were similar (Kaur et al., 2008). Research also demonstrated that KO of the orexin system results in narcolepsy and sleep fragmentation (Coffey et al., 2021). These results suggest that orexin neurons may sense the metabolic status and modulate arousal to promote food-seeking behavior during RF regimes. The exact role of orexin neurons in the metabolic balance and sleep-wake regulation during RF in aged mice needs to be elucidated in the future.

At present, there is little consistency in RF protocols in animal studies. For example, in many animal studies on RF, food was provided during the inactive period when animals spend more time sleeping. In other studies, food was provided during the active period. Moreover, the feeding window has also varied from 2 to 8 h per day in different animal studies (Northeast et al., 2020; Caba et al., 2021). An inappropriate RF protocol can result in erroneous conclusions (Pendergast and Yamazaki, 2018). A standardized and optimized experimental protocol for RF is needed to elucidate the mechanism of RF in improving metabolic health and sleep-wake cycles.

The present study has some limitations. As sleep-wake cycles vary with age and gender (Dorsey et al., 2020), one limitation is that our study included only aged male mice. Thus, it was not possible to compare sex- and age-related differences in the RF regime. Another limitation of the study was that although the RF regime was associated with benefits in the aging mice, such a regime does not resemble RF in humans because the sleep structure and food consumption behavior of humans and rodents differ greatly. This is a problem common to all RF studies that utilize animal models.

## Conclusion

In summary, food restriction in aged mice had a profound impact on body weights, blood glucose and cholesterol levels, and amylopsin and lipase activities. In addition, RF changed the sleep-wake profiles of the mice, with an increase in the mean duration of wakefulness and a decrease in the number of wakefulness episodes. Food restriction also influenced sleep and wakefulness quality during the 2-h period prior to food availability. Our results indicate that RF during aging may prohibit or delay the onset of age-related diseases by improving metabolic health, without severe deleterious effect on sleep. They also suggest that RF could be a novel, non-pharmacological intervention to prevent metabolic disorders in the elderly population.

## Data Availability Statement

The original contributions presented in the study are included in the article/Supplementary Material, further inquiries can be directed to the corresponding authors.

## Ethics Statement

The animal study was reviewed and approved by the Animal Care and Use Committee of Anhui Medical University.

## Author Contributions

QX, Y-XX, and M-KZ conceived and designed the study. Y-XX, G-YL, H-QB, S-CW, W-HC, and G-JC carried out the experiments. QX, G-YL, P-PZ, and QJ analyzed the data. C-BG, M-KZ, G-HC, D-FZ, and QX wrote the manuscript. All authors contributed to the study and approved the final version.

## Conflict of Interest

The authors declare that the research was conducted in the absence of any commercial or financial relationships that could be construed as a potential conflict of interest.

## Publisher’s Note

All claims expressed in this article are solely those of the authors and do not necessarily represent those of their affiliated organizations, or those of the publisher, the editors and the reviewers. Any product that may be evaluated in this article, or claim that may be made by its manufacturer, is not guaranteed or endorsed by the publisher.

## Supplementary Material

Supplementary Figure 1 Time the mice in the RF and CTRL groups spent in sleep-wake stages on day 3 of the RF protocol. Time course of wakefulness **(A)**, NREM sleep **(B)**, and REM sleep on day 14 of the RF protocol **(C)**. Cumulative time of wakefulness, NREM sleep, and REM sleep during the light period **(D)**, dark period **(E)**, and 24-h period **(F)**. The gray box indicates food availability during the dark period. Open and closed bars above the *x*-axis indicate light and dark periods, respectively. ^∗∗^*p* < 0.01 versus CTRL, *n* = 6. CTRL, control; RF, restricted feeding.

Supplementary Figure 2 Time the mice in the RF and CTRL groups spent in sleep-wake stages on day 7 of the RF protocol. Time course of wakefulness **(A)**, NREM sleep **(B)**, and REM sleep on day 14 of the RF protocol **(C)**. Cumulative time of wakefulness, NREM sleep, and REM sleep during the light period **(D)**, dark period **(E)**, and 24-h period **(F)**. The gray box indicates food availability during the dark period. Open and closed bars above the *x*-axis indicate light and dark periods, respectively. ^∗∗^*p* < 0.01 versus CTRL, *n* = 6. CTRL, control; RF, restricted feeding.

Supplementary Figure 3 Sleep-wake architecture in the RF and CTRL groups on day 0 of the RF protocol. The mean durations of wakefulness, NREM sleep, REM sleep **(A)**, and number/type of episode during each stage **(B)** during the 10 h of the light period (08:00–18:00). **(C)** Transitions between stages during the 10 h of the light period, *n* = 6. CTRL, control; RF, restricted feeding; R, REM sleep; S, slow-wave sleep; W, wakefulness.

Supplementary Figure 4 Sleep-wake architecture in the RF and CTRL groups on day 14 of the RF protocol. The mean durations of wakefulness, NREM sleep, REM sleep **(A)**, and number/type of episode during each stage **(B)** during the 10 h of the light period (08:00–18:00). **(C)** Transitions between stages during the 10 h of the light period, *n* = 6. CTRL, control; RF, restricted feeding; R, REM sleep; S, slow-wave sleep; W, wakefulness.

Supplementary Figure 5 Characteristics of EEG power density during wakefulness, NREM sleep, and REM sleep in the 10 h during the light period (08:00–18:00). EEG power density during wakefulness **(A)**, NREM sleep **(B)**, and REM sleep **(C)** in the 10 h during the light period under the baseline condition (day 0 of the RF regime), *n* = 6. CTRL, control; RF, restricted feeding.

Supplementary Figure 6 Characteristics of EEG power density during wakefulness, NREM sleep, and REM sleep in the 10 h during the light period (08:00–18:00). EEG power density during wakefulness **(A)**, NREM sleep **(B)**, and REM sleep **(C)** in the 10 h during the light period on day 14 of the RF regime, *n* = 6. CTRL, control; RF, restricted feeding.
