# Supplementary figures and images for: Effect of Restricted Feeding on Metabolic Health and Sleep-Wake Rhythms in Aging Mice
Source: Front Neurosci. 2021 Sep 7;15:745227. doi: 10.3389/fnins.2021.745227 (PMC8453873; doi:10.3389/fnins.2021.745227)

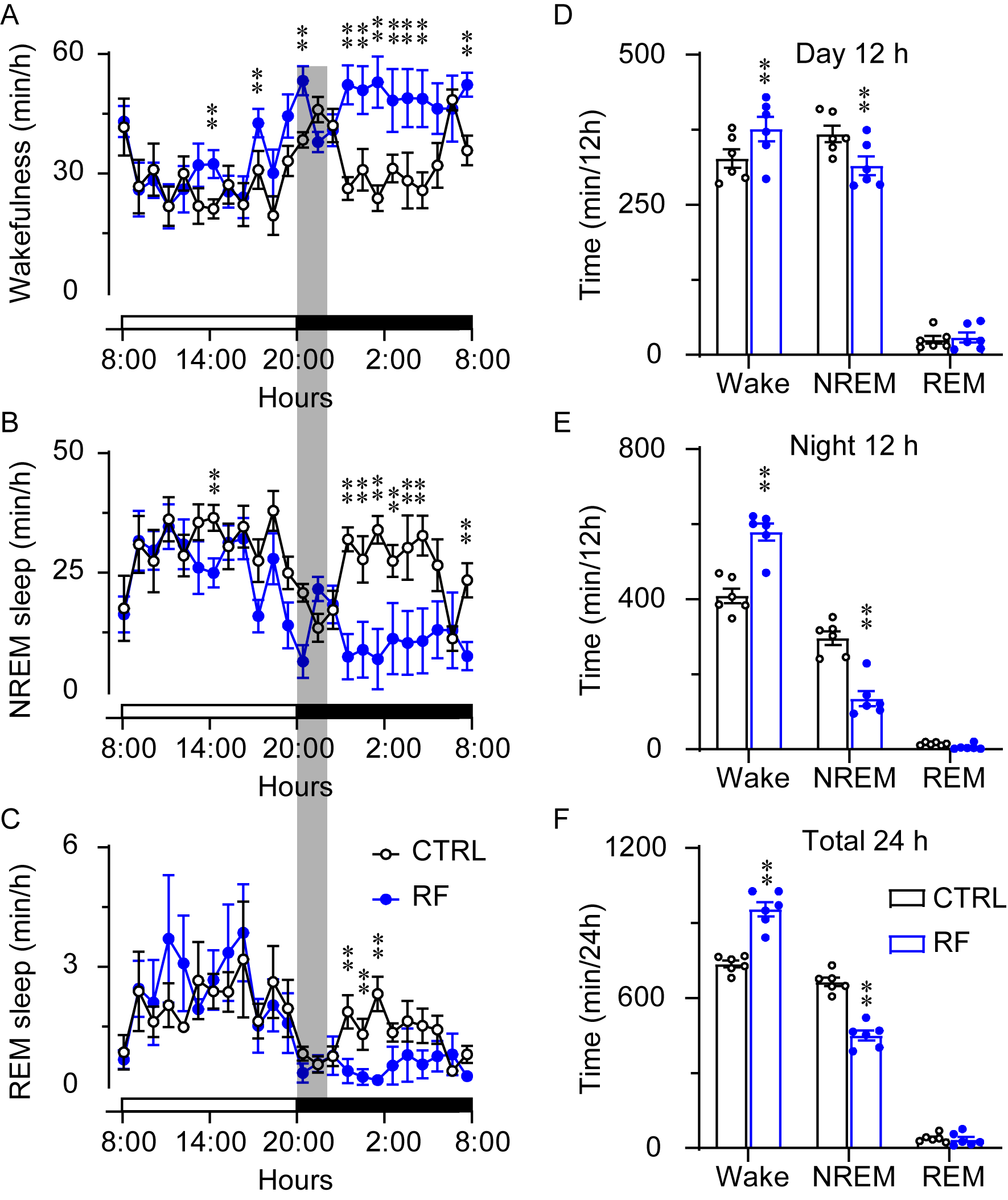

Supplement: Supplementary Figure 1 — Time the mice in the RF and CTRL groups spent in sleep-wake stages on day 3 of the RF protocol. Time course of wakefulness (A), NREM sleep (B), and REM sleep on day 14 of the RF protocol (C). Cumulative time of wakefulness, NREM sleep, and REM sleep during the light period (D), dark period (E), and 24-h period (F). The gray box indicates food availability during the dark period. Open and closed bars above the x-axis indicate light and dark periods, respectively. ∗∗p < 0.01 versus CTRL, n = 6. CTRL, control; RF, restricted feeding. [file Image_1.TIF]

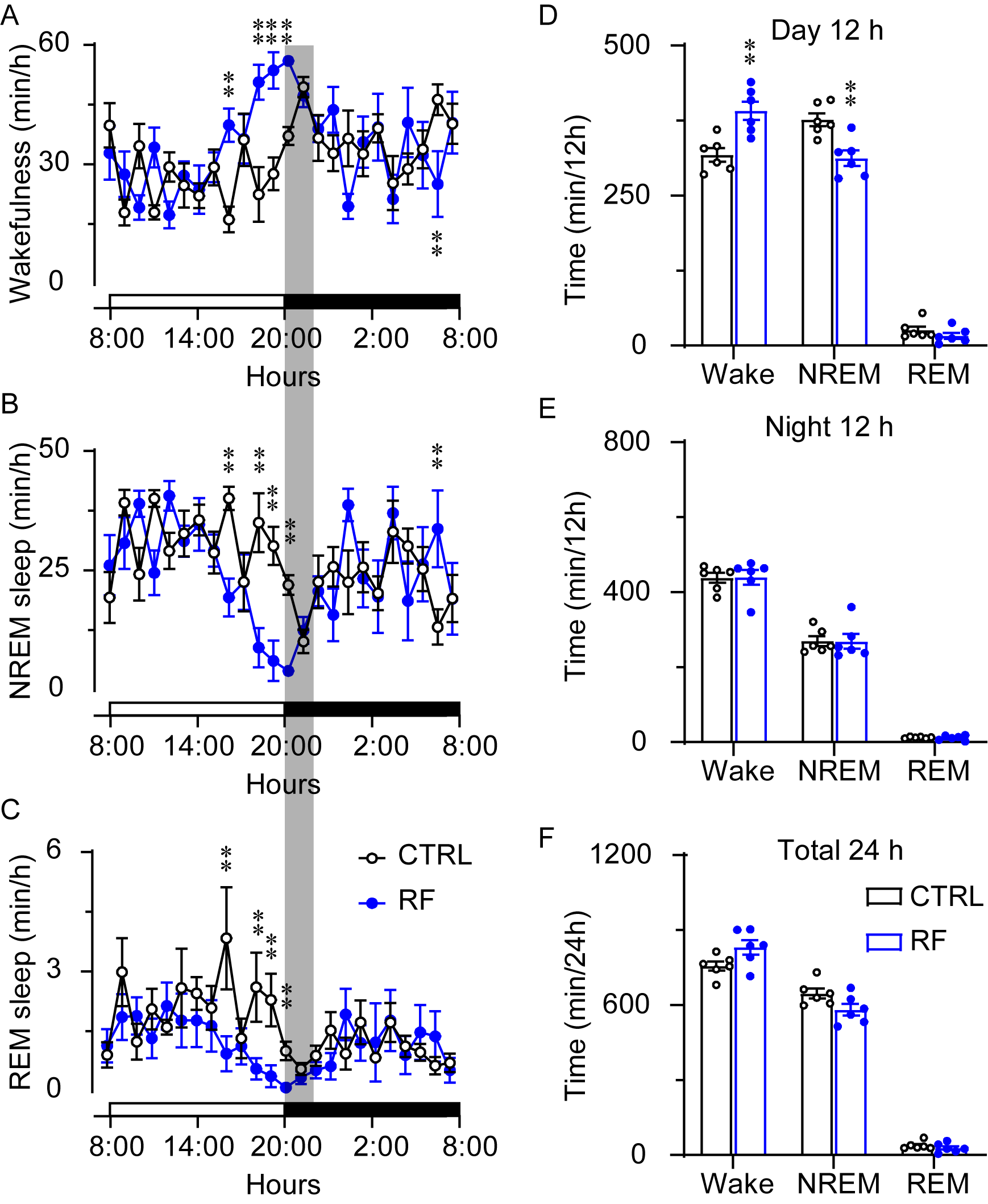

Supplement: Supplementary Figure 2 — Time the mice in the RF and CTRL groups spent in sleep-wake stages on day 7 of the RF protocol. Time course of wakefulness (A), NREM sleep (B), and REM sleep on day 14 of the RF protocol (C). Cumulative time of wakefulness, NREM sleep, and REM sleep during the light period (D), dark period (E), and 24-h period (F). The gray box indicates food availability during the dark period. Open and closed bars above the x-axis indicate light and dark periods, respectively. ∗∗p < 0.01 versus CTRL, n = 6. CTRL, control; RF, restricted feeding. [file Image_2.TIF]

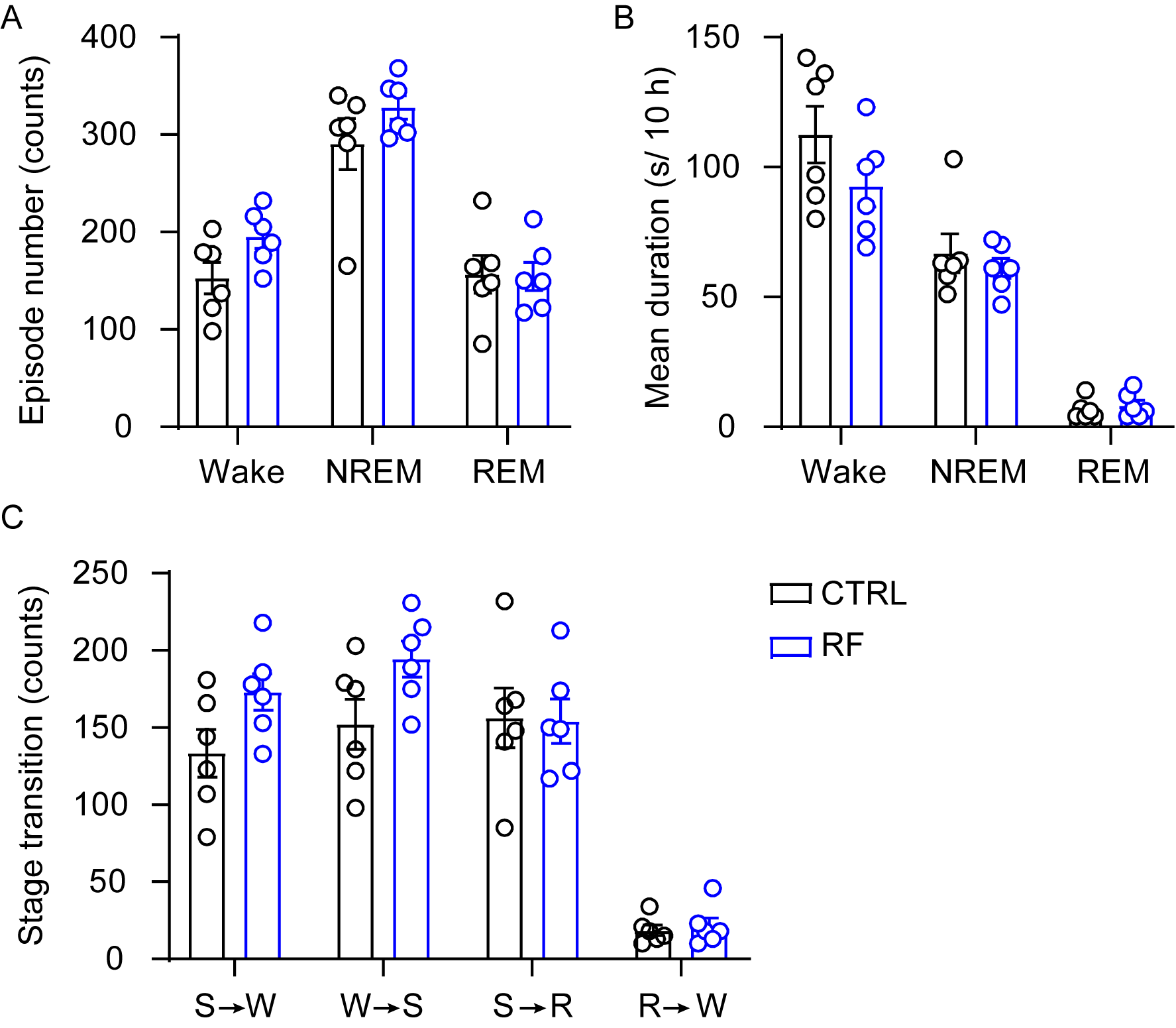

Supplement: Supplementary Figure 3 — Sleep-wake architecture in the RF and CTRL groups on day 0 of the RF protocol. The mean durations of wakefulness, NREM sleep, REM sleep (A), and number/type of episode during each stage (B) during the 10 h of the light period (08:00–18:00). (C) Transitions between stages during the 10 h of the light period, n = 6. CTRL, control; RF, restricted feeding; R, REM sleep; S, slow-wave sleep; W, wakefulness. [file Image_3.tif]

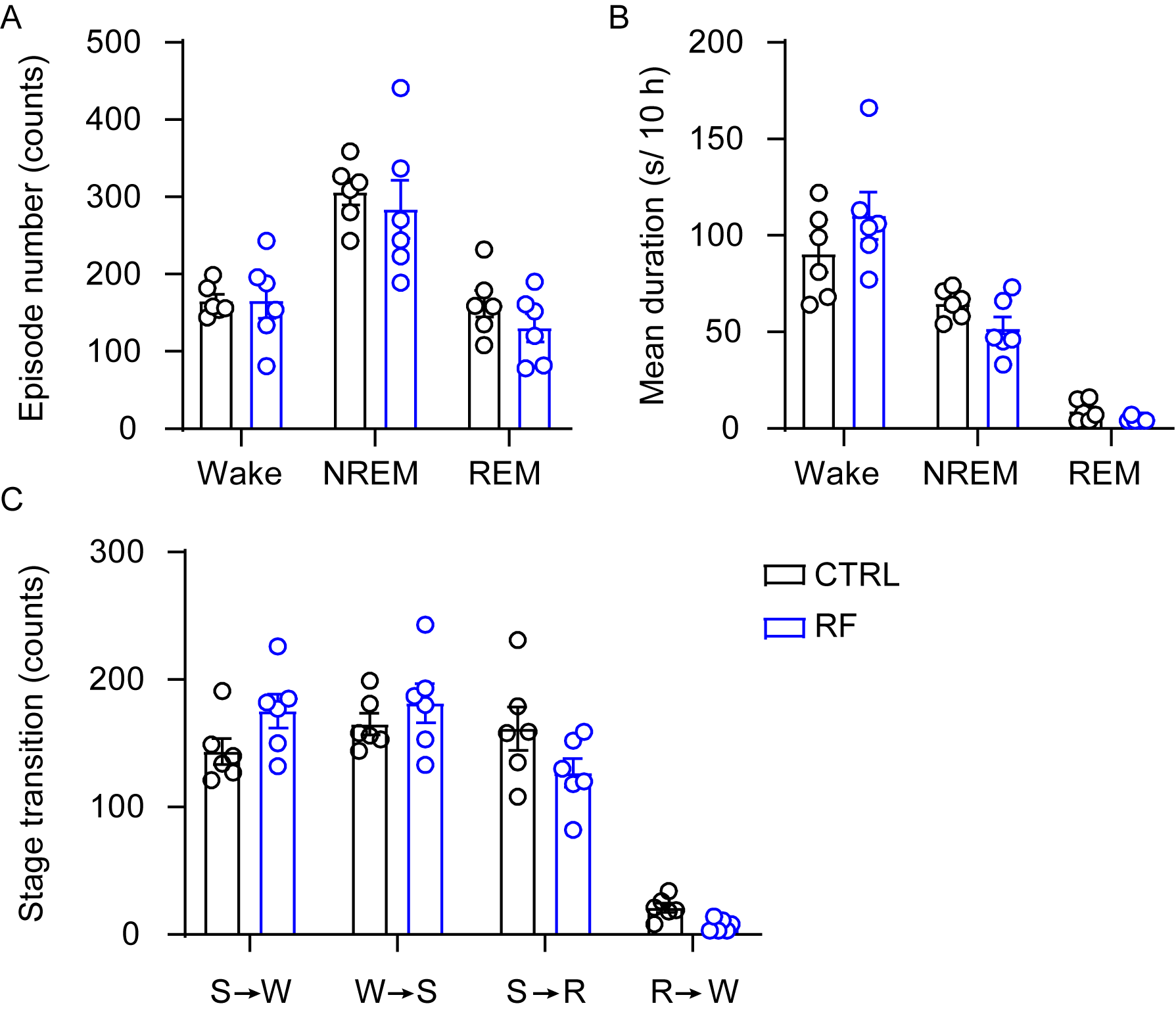

Supplement: Supplementary Figure 4 — Sleep-wake architecture in the RF and CTRL groups on day 14 of the RF protocol. The mean durations of wakefulness, NREM sleep, REM sleep (A), and number/type of episode during each stage (B) during the 10 h of the light period (08:00–18:00). (C) Transitions between stages during the 10 h of the light period, n = 6. CTRL, control; RF, restricted feeding; R, REM sleep; S, slow-wave sleep; W, wakefulness. [file Image_4.tif]

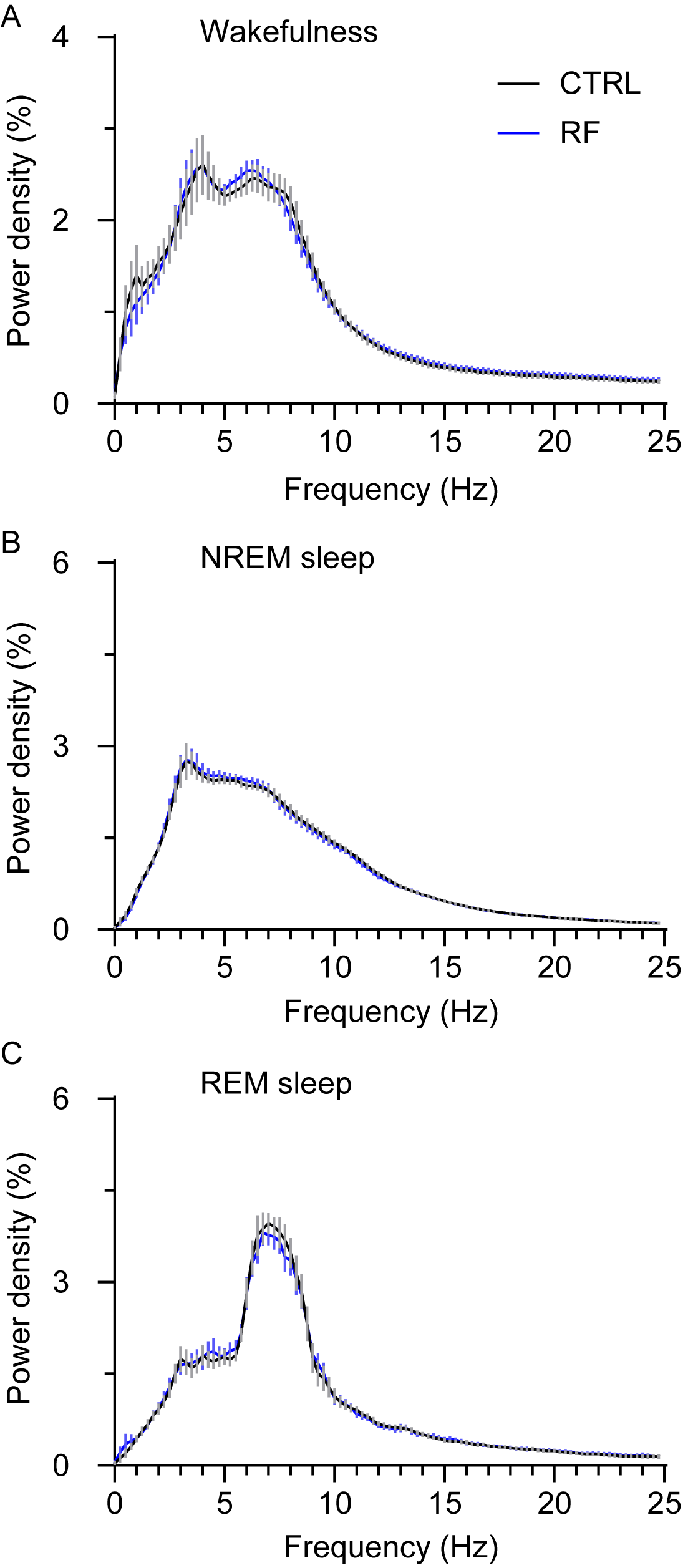

Supplement: Supplementary Figure 5 — Characteristics of EEG power density during wakefulness, NREM sleep, and REM sleep in the 10 h during the light period (08:00–18:00). EEG power density during wakefulness (A), NREM sleep (B), and REM sleep (C) in the 10 h during the light period under the baseline condition (day 0 of the RF regime), n = 6. CTRL, control; RF, restricted feeding. [file Image_5.tif]

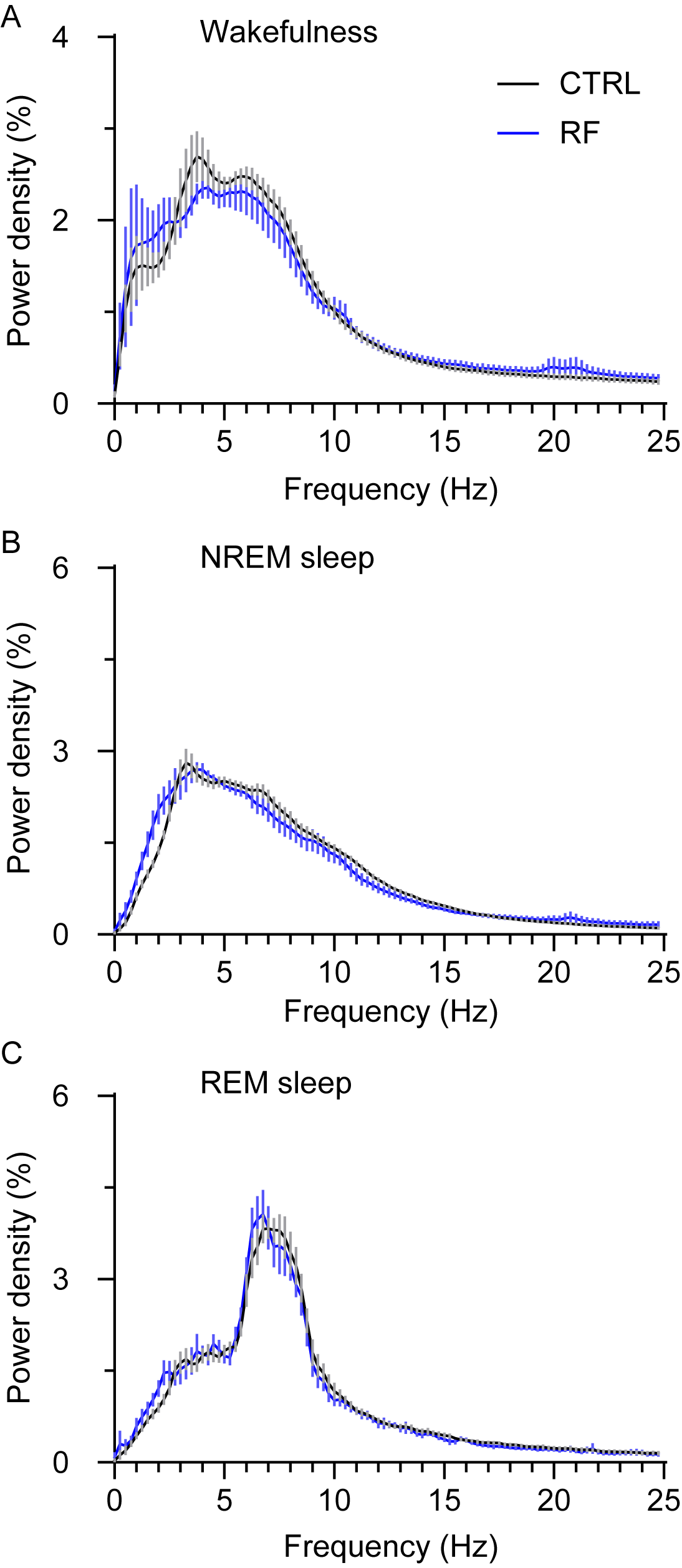

Supplement: Supplementary Figure 6 — Characteristics of EEG power density during wakefulness, NREM sleep, and REM sleep in the 10 h during the light period (08:00–18:00). EEG power density during wakefulness (A), NREM sleep (B), and REM sleep (C) in the 10 h during the light period on day 14 of the RF regime, n = 6. CTRL, control; RF, restricted feeding. [file Image_6.tif]
